# Bacterial community succession and functional gene dynamics of nitrogen and phosphorus cycling in recirculating aquaculture systems of shrimp

**DOI:** 10.1093/femsec/fiag081

**Published:** 2026-07-25

**Authors:** Arslan Emmanuel, Muhammad Naeem Ramzan, Zhongming Zheng

**Affiliations:** School of Marine Sciences, Ningbo University, Ningbo 315211, China; School of Marine Sciences, Ningbo University, Ningbo 315211, China; School of Marine Sciences, Ningbo University, Ningbo 315211, China

**Keywords:** RAS, functional genes, nitrogen cycle, phosphorus cycle, water, biofilm

## Abstract

Recirculating aquaculture systems (RAS) rely heavily on microbial processes to remove harmful nitrogenous compounds such as ammonia and nitrite. Microbial assemblages within these systems also contribute substantially to broader biogeochemical cycling. Gaining a detailed understanding of the functional potential and abundance of microorganisms responsible for nitrogen (N) and phosphorus (P) transformations is therefore crucial for evaluating nutrient remediation efficiency. In this study, we integrated high-throughput 16S rRNA sequencing with a GeoChip functional gene array to investigate how microbial community dynamics correspond to nutrient fluctuations in full-scale RAS. A suite of multivariate statistical approaches, including ordination and network-based analyses, was applied to characterize shifts in both taxonomic structure and functional gene profiles across operational phases. Our results revealed clear temporal patterns in functional gene abundance. Denitrification-related genes (*nosZ, napA, nirK/S*) became more prominent toward the later stages of operation, while nitrification genes (*amoA/B*) were most abundant early in the cycle and declined thereafter. Genes involved in ammonification (*ureC*) and several P-cycling genes (*phnK, phoD, phoX*) also increased notably during the final phase. These trends underscore the close relationship between bacterial community succession, species turnover, and nutrient cycling capacity. Overall, the study enhances current understanding of functional microbial dynamics in RAS and provides insights that could guide the development of improved nutrient management and bioremediation strategies for aquaculture systems.

## Teaser

This study shows how beneficial microbes change over time in shrimp recirculating aquaculture systems, driving nitrogen and phosphorus removal and helping improve water quality and sustainability.

## Introduction

In high-intensity mariculture operations, uneaten feed and metabolic wastes release considerable quantities of nitrogen-rich compounds and bioavailable phosphate into the water column. These accumulated nutrients can become harmful to cultured species and may also degrade the ecological quality of adjacent environments (Li et al. 2023). Pollution resulting from aquaculture activities has thus become a major environmental concern, posing significant challenges for water quality management and ecosystem protection (Wang et al. 2018b). Consequently, the development of efficient wastewater treatment strategies is essential for the effective removal of these contaminants (Kim et al. 2020). In recent years, RAS have gained prominence as eco-friendly, water-efficient and high-yield alternatives for intensive aquaculture production (Ahmed and Turchini 2021). However, the accumulation of toxic nitrogenous compounds particularly ammonium and nitrite remains a major limitation to the optimal performance of RAS (Shao et al. 2019). Effective removal of these nitrogen pollutants is therefore critical for maintaining water quality and ensuring system sustainability. Nevertheless, achieving efficient nitrogen removal in aquaculture wastewater remains challenging due to its inherently low organic carbon content and high levels of dissolved oxygen (DO), which constrain microbial denitrification processes (Shao et al. 2019).

Culture-independent molecular approaches have greatly advanced the study of microbial communities by enabling detailed assessment of their taxonomic diversity, functional capabilities, and responses to environmental change (Zhou et al. 2015, Deng et al. 2016, Feng et al. 2017). Techniques such as high-density 16S rRNA oligonucleotide microarrays (e.g. PhyloChip) and next-generation 16S rRNA gene sequencing are now routinely applied to characterize shifts in microbial community composition and structure (Hazen et al. 2010). More comprehensive insights into microbial functionality can be achieved through metagenomic and metatranscriptomic sequencing, which together allow the detection of novel genes, regulatory pathways, and metabolic traits (Weinstock 2012). Functional microarrays such as GeoChip further complement these methods by providing highly specific detection of genes involved in key biogeochemical cycles (Tu et al. 2014).

Both sequencing-based and microarray-based tools have become central to microbial ecological studies because they offer extensive coverage of functional and taxonomic markers. These datasets are typically analyzed using relative abundance metrics, which describe how specific taxa or genes are proportionally represented within a community. While useful for identifying compositional shifts among samples, relative abundance does not capture changes in the total size or absolute metabolic potential of microbial populations. Quantifying absolute abundance expressed as gene copy numbers or transcript counts is therefore essential for accurately interpreting microbial contributions to ecosystem processes. Incorporating absolute gene quantification has been shown to substantially improve predictions of nitrogen transformation dynamics compared with analyses based solely on community diversity (Graham et al. 2016). Quantitative PCR (qPCR) remains one of the most reliable tools for determining absolute gene copy numbers, as demonstrated by studies linking *amoA* gene abundance with ammonium concentrations in marine nitrification environments (Wuchter et al. 2006). Other investigations have similarly employed the abundances of *amoA, nirK/nirS, nosZ*, and related functional genes to evaluate spatial or environmental variations in nitrification and denitrification potential (Petersen et al. 2012).

Microorganisms underpin global biogeochemical cycles and play indispensable roles in carbon and sulfur turnover, organic matter degradation, nitrogen transformations, and phosphorus mobilization (van der Heijden et al. 2008, Zarraonaindia et al. 2013, Vanwonterghem et al. 2014). Understanding their community structure, functional gene diversity, and quantitative gene profiles is critical for elucidating how microbial processes respond to environmental pressures at both local and global scales (Penuelas et al. 2013, Graham et al. 2016). Within the nitrogen cycle, nitrification is particularly important in engineered and natural aquatic systems, including RAS, because it prevents the accumulation of toxic reduced nitrogen compounds (Camargo and Alonso 2006). This process proceeds through the oxidation of ammonia to nitrite and then to nitrate, mediated by ammonia-oxidizing bacteria and archaea (AOB/AOA), nitrite-oxidizing bacteria (NOB), or complete ammonia oxidizers (comammox). In RAS biofilters, these nitrifying microorganisms colonize biofilm substrates alongside diverse heterotrophic populations, collectively forming complex communities with high functional redundancy (van Kessel et al. 2010, Ruiz et al. 2019).

Ammonia-oxidizing bacteria in RAS are typically represented by *Nitrosomonas* and *Nitrosospira*, while *Nitrosococcus* is common in marine systems (Keuter et al. 2017). Ammonia-oxidizing archaea, well adapted to low-nutrient conditions, also occur frequently in marine or brackish RAS (Huang et al. 2018). Nitrite oxidation is performed by several NOB lineages (Daims et al. 2016), including *Nitrospira, Nitrobacter, Nitrococcus*, and *Nitrospina*, with additional uncultured clades detected through metagenomic studies (Ngugi et al. 2016, Keuter et al. 2017). Although *Nitrotoga* is only sporadically reported in RAS (Hüpeden et al. 2016, Keuter et al. 2017), recent discoveries have demonstrated that some *Nitrospira* species can carry out complete ammonia oxidation (Daims et al. 2015, van Kessel et al. 2015), challenging the traditional two-step model of nitrification and broadening our understanding of nitrogen transformations in RAS (Bartelme et al. 2017). In contrast to nitrogen cycling, the functional genomics of phosphorus transformations remains less well characterized. Commonly studied P-cycling genes include polyphosphate metabolism genes (*ppx, ppk*) and *phytase*, while genes involved in inorganic phosphate solubilization or C–P bond cleavage are less frequently quantified due to limited primer availability (He et al. 2010). To overcome these constraints, the present study (i) developed a set of targeted primers for key P-cycling genes and (ii) designed a high-throughput qPCR-based Geochip functional gene array capable of simultaneously quantifying nitrogen and phosphorus related genes. This integrated approach provides a more comprehensive assessment of the microbial functional potential associated with N and P cycling and enhances our ability to evaluate microbial responses to environmental variation in RAS.

## Material and methods

### Experimental design

This study was carried out at Jufu agricultural farm, located in the Beilun district of Ningbo, Zhejiang province, China. The experiment utilized four independent RAS, each comprising four culture tanks with a working volume of 38 m³. Each RAS was equipped with its own recirculation pump, a mechanical filtration component, and a 40 m³ bio-filter operating under a moving bed biofilm reactor (MBBR) design (Emmanuel et al. 2025a). All units were maintained within a “greenhouse” facility to minimize environmental fluctuations and ensure stable temperature conditions throughout the culture period. On May 1, 2023, juvenile *Litopenaeus vannamei* were stocked at a density of 25 000 folks per tank, equal to ∼658 shrimp m⁻³. Salinity in all systems was maintained at ∼5 ppt for the entire rearing duration. The culture phase extended for ∼3 months, ending on 18 July 2023. Shrimp were fed a commercial pelleted diet containing 48% crude protein (Table A.2) three times daily at 09:00 am, 2:00 pm and 8:00 pm. Initial feeding rates were set at 6% of estimated biomass and subsequently modified based on feeding activity and observed consumption. To evaluate nitrogen flow and transformation processes within each system, data were collected on feed inputs, uneaten feed residues, fecal waste output, water exchange volumes and the concentrations of major nitrogen species. Environmental parameters such as water temperature and transparency were monitored daily before feeding to document system conditions and to assess their potential influence on microbial activity associated with nitrogen cycling.

### Sampling and water physicochemical analyses

Specimens were collected on Days 15, 31, 46, 61, and 76 to capture the major developmental phases of *L. vannamei* throughout the production cycle. For each bio-filter, four sampling points were chosen randomly to ensure representative spatial coverage. At every point, water samples, biofilm material, and shrimp samples were collected, resulting in a total of 80 biological samples. All sampling activities were conducted at 09:00 am to minimize diurnal fluctuations and maintain consistency in the assessment of microbial community changes. Shrimp wet weights were recorded at the beginning of the trial (0.02 ± 0.005 g) and upon completion on Day 90 (13.89 ± 2.71 g) prior to transport for laboratory processing (Table A.1). Over the entire rearing period, shrimp exhibited an average daily growth rate of 0.154 g, a feed conversion ratio (FCR) of 1.81, and a survival rate of 72.21% (Table A.3). For bacterial community characterization, water and biofilm samples were filtered through sterile 0.22 µm membrane filters, whereas sediment collected from the tank bottoms was immediately stored at −80°C prior to DNA extraction. Water destined for nutrient analysis was filtered using 0.45 µm polycarbonate membranes and frozen at −20°C until further processing. Daily monitoring of key water quality variables including temperature, salinity, pH, and dissolved oxygen was performed using a multi-parameter probe. In addition, nutrient concentrations were measured weekly using a SmartChem 450 automated analyzer (KPM Analytics, Westborough, MA, USA). Parameters assessed included COD_Mn_, soluble phosphate (PO₄³⁻-P), nitrite (NO₂⁻-N), nitrate (NO₃⁻-N), ammonia nitrogen (NH₄⁺-N), total nitrogen (TN), and total phosphorus (TP). Standard analytical procedures were followed for all chemical determinations: ammonia was analyzed via the hypobromite oxidation method; nitrate was quantified after reduction with a zinc–cadmium column followed by spectrophotometric reading (Ramzan et al. 2025a); nitrite was measured using the naphthyl ethylenediamine colorimetric assay; and phosphate was quantified using the phosphomolybdenum blue method (Emmanuel et al. 2025a). TN and TP were determined using potassium persulfate oxidation (Ramzan et al. 2025b), while COD_Mn_ was measured with a modified alkaline permanganate protocol followed by titration using potassium permanganate (Emmanuel et al. 2025b).

### Bacterial DNA extraction, Illumina HiSeq sequencing, and bioinformatics analysis

Genomic DNA was isolated from shrimp-derived samples using a commercial extraction Minkgene DNA kit (Guangdong Magigene Biotechnology Co., Ltd., Guangzhou, China) following the manufacturer’s recommended protocol (Emmauel et al. 2025b). DNA concentration and purity were measured using a NanoDrop One spectrophotometer (Thermo Fisher Scientific, Massachusetts, USA) to ensure that the extracts met the requirements for high-throughput sequencing. The bacterial community was characterized by amplifying the V3–V4 region of the 16S rRNA gene using the universal primers (338F and 806R). Amplified products were subjected to paired-end sequencing (2 × 250 bp) on an Illumina HiSeq 2500 platform operated by Guangdong Magigene Biotechnology Co., Ltd. (Guangzhou, China). Initial sequence processing was carried out with the USEARCH software suite (version 11.0.667_I8). Reads were first filtered to remove low-quality bases, sequencing artifacts, and chimeric sequences using the UNOISE3 denoising workflow. Default parameters were applied except for adjustments to the minimum read size (minsize = 8) and the unoise_alpha setting (alpha = 2). The resulting high-quality reads were then resolved into zero-radius operational taxonomic units (ZOTUs), enabling fine-scale profiling of bacterial variants. Taxonomic assignment of representative ZOTU sequences was performed using the SILVA SSU Ref NR 99 database (version 138.01). This approach ensured consistent cataloging across bacterial taxa and provided a robust foundation for subsequent analyses of community composition, diversity, and ecological patterns.

### Detection of nitrogen and phosphorus, functional gene using Geochip

After sample collection, bacterial DNA extracts were then aliquoted into a 384-well sample plate together with the required quantitative PCR (qPCR) reaction components. In parallel, an assay source plate containing primer mixtures and qPCR master reagents was prepared in an identical 384-well format. Using an automated microsampling system, reaction mixtures from both plates were dispensed into the nanowells of a high-throughput GeoChip qPCR array. Amplification and real-time fluorescence detection were performed using the SmartChip Real-Time PCR platform, which generated amplification and melting curves automatically (Nolan et al. 2006). The GeoChip platform utilized highly specific oligonucleotide probes designed from conserved regions of functional genes involved in biogeochemical cycling, enabling the detection of a broad range of nitrogen and phosphorus cycling genes across diverse microbial taxa. Probe specificity is achieved through rigorous in silico design and validation to minimize non-specific hybridization, although some degree of cross-hybridization among closely related gene sequences may occur. The detection sensitivity of the system is dependent on DNA quality and amplification efficiency, with reliable detection generally achieved within a defined cycle threshold (Ct) range. Gene detection outcomes and corresponding cycle threshold (Ct) values were processed using the Canco analysis platform. To estimate the relative abundance of functional genes, Ct values for each target were standardized against those of the bacterial 16S rRNA gene, which served as an alternative for total bacterial abundance. Given the variability in 16S rRNA gene copy number among taxa, the resulting values were interpreted as relative estimates rather than absolute abundances, which were used as the internal reference for normalization. Absolute quantities of the 16S rRNA gene were determined through a standard curve–based calibration procedure following the Roche quantitative framework. Absolute copy numbers for all remaining functional genes were then derived by converting normalized values according to the method outlined by Mardis and McCombie (2017), enabling quantitative comparison of gene abundances across samples. This approach allows for high-throughput, sensitive, and relatively specific detection of functional genes; however, it is important to note that detection limits may vary depending on gene abundance and environmental sample complexity.

### Statistical data analyses

Nutrient content data were analyzed using one-way ANOVA followed by Tukey’s post hoc test to identify statistically significant differences between groups. Variations in gene abundances were evaluated using IBM SPSS Statistics 20, while data visualization was performed with GraphPad Prism 10.0 and R software (version 4.3.2). Absolute quantities of functional genes were charted in Microsoft Excel, and star plots were generated using the ‘ggplot2’ package in R. Group-specific differences in microbial and gene abundance profiles were evaluated using the Kruskal–Wallis nonparametric test, with pairwise Wilcoxon rank-sum tests applied to verify the robustness of detected differences (Chang et al. 2022). Bubble plots illustrating shifts in relative gene abundance were visualized using SR Plot software. Correlations between microbial taxa and functional gene abundances were computed using the “Hmisc” package in R. Co-occurrence network structures were constructed with the ‘igraph’ package and subsequently visualized in Gephi (version 0.10.1), enabling integrative assessment of microbial–functional gene interactions.

## Results

### The bacterial community composition and relationship of functional genes to nitrogen and phosphorus in water and biofilm

**Figure 1:**
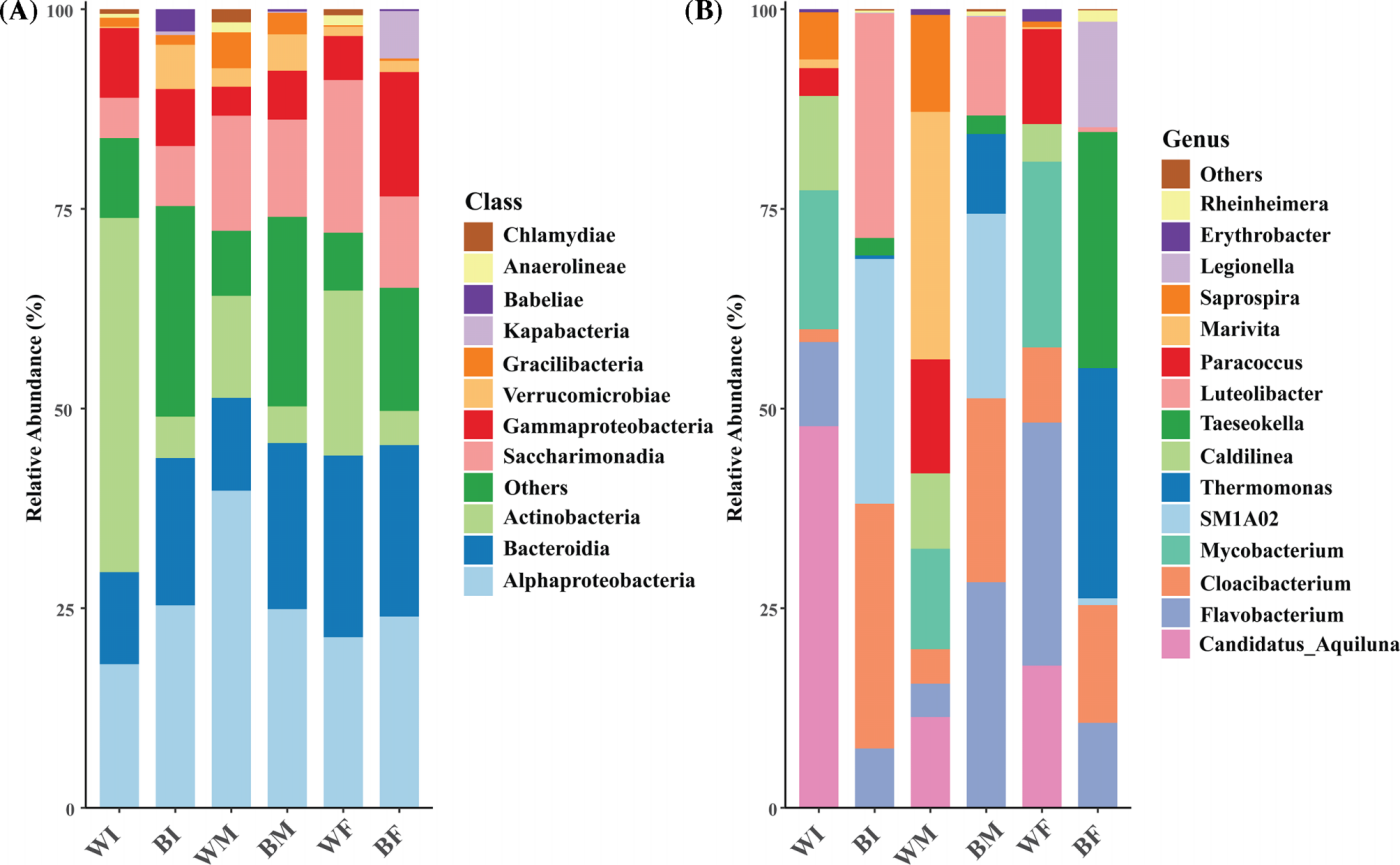
Relative abundance of the top 10 dominant bacterial taxa in water and biofilm samples across different time points, shown at the class level (A) and genus level (B). *WI* water initial, *BI* biofilm initial, *WM* water middle, *BM* biofilm middle, *WF* water final, *BF* biofilm final.

The class-level analysis revealed dominant bacterial community composition in the water and biofilm column across three successional phases (initial, middle and final) included Alphaproteobacteria, Actinobacteria, Bacteroidia, Saccharimonadia, and Gammaproteobacteria. In the water column, Alphaproteobacteria were most abundant during the middle phase, whereas in the biofilm they were initially dominant and subsequently showed a slight decline in the final phase. Furthermore, Actinobacteria, the second most abundant class, showed a significant increase during the initial phase, followed by a decline in the final phase in both the water column and biofilm. The third most abundant class was Bacteroidia which was significantly increased in the final phase in water as well as in the biofilm (Fig. 1A). At the genus level, *Candidatus Aquiluna* and *Cloacibacterium* were the most abundant during the initial phase. *Flavobacterium* exhibited significantly higher abundance in the middle phase of the biofilm, whereas in the water column it was more prominent during the final phase. In the final phase, *Mycobacterium* and *Cloacibacterium* dominated in the water column, while *Taeseokella* and *Thermomonas* were the most prevalent genera in the biofilm (Fig. 1B).

**Figure 2:**
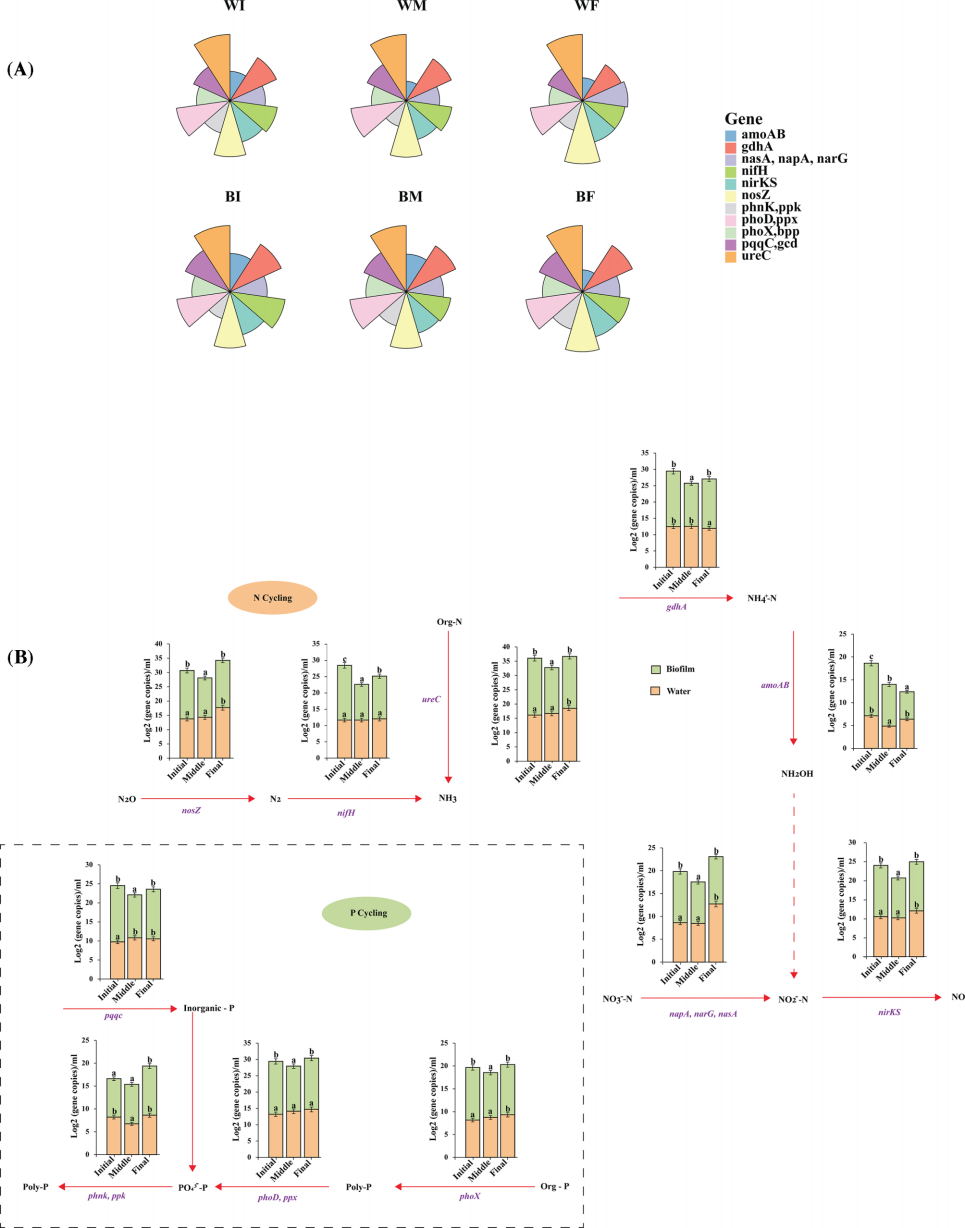
Absolute quantification of nitrogen (N) and phosphorus (P) cycling genes in the water column and biofilm. Star-map visualization of functional gene abundances in the water column and biofilm (A). Circulation pathways representing gene interaction networks in the water column and biofilm (B). Different letters indicate significant differences among the initial, middle, and final stages within the same habitat (*P* < 0.05, one-way ANOVA; Tukey’s HSD).

Functional genes associated with nitrogen (N) and phosphorus (P) metabolisms are directly linked to nutrient cycling processes. Using the GeoChip technique, a total of 31 key genes involved in N and P cycling were identified, 22 related to nitrogen and 9 to phosphorus. These genes encompass various biogeochemical pathways, including aerobic ammoxidation, organic nitrogen mineralization, nitrification, anaerobic ammonium oxidation (anammox), denitrification, nitrogen fixation, and ammonification for nitrogen, as well as organic phosphorus mineralization and other phosphorus cycling mechanisms (Fig. 2). In the water column, the abundances of key denitrification genes *nosZ, napA*, and *nirK/S* were significantly higher in the final stage compared to the biofilm (*P* < 0.05). The nitrogen fixation gene *nifH*, which facilitates the conversion of atmospheric N₂ to NH₃, exhibited significantly higher abundance in the biofilm during the initial phase, followed by a decline in the final stage. The *gdhA* gene, involved in the conversion of organic nitrogen to ammonium (NH₄⁺-N), showed a significant increase in the biofilm during the initial phase, with a slight decrease observed by the final phase (p < 0.05). A notable rise in *ureC* abundance, associated with urea metabolism, was also recorded in the final phase of both the water column and biofilm (*P* < 0.05). The abundances of *ppx* and *phnK*, key genes involved in organic phosphorus mineralization, increased in the final phase of the biofilm. Moreover, *phoD* showed a significant rise in abundance during the final phase in both the water column and biofilm (*P* < 0.05).

### Phase and habitat-specific variation in nitrogen and phosphorus cycling genes abundance

**Figure 3:**
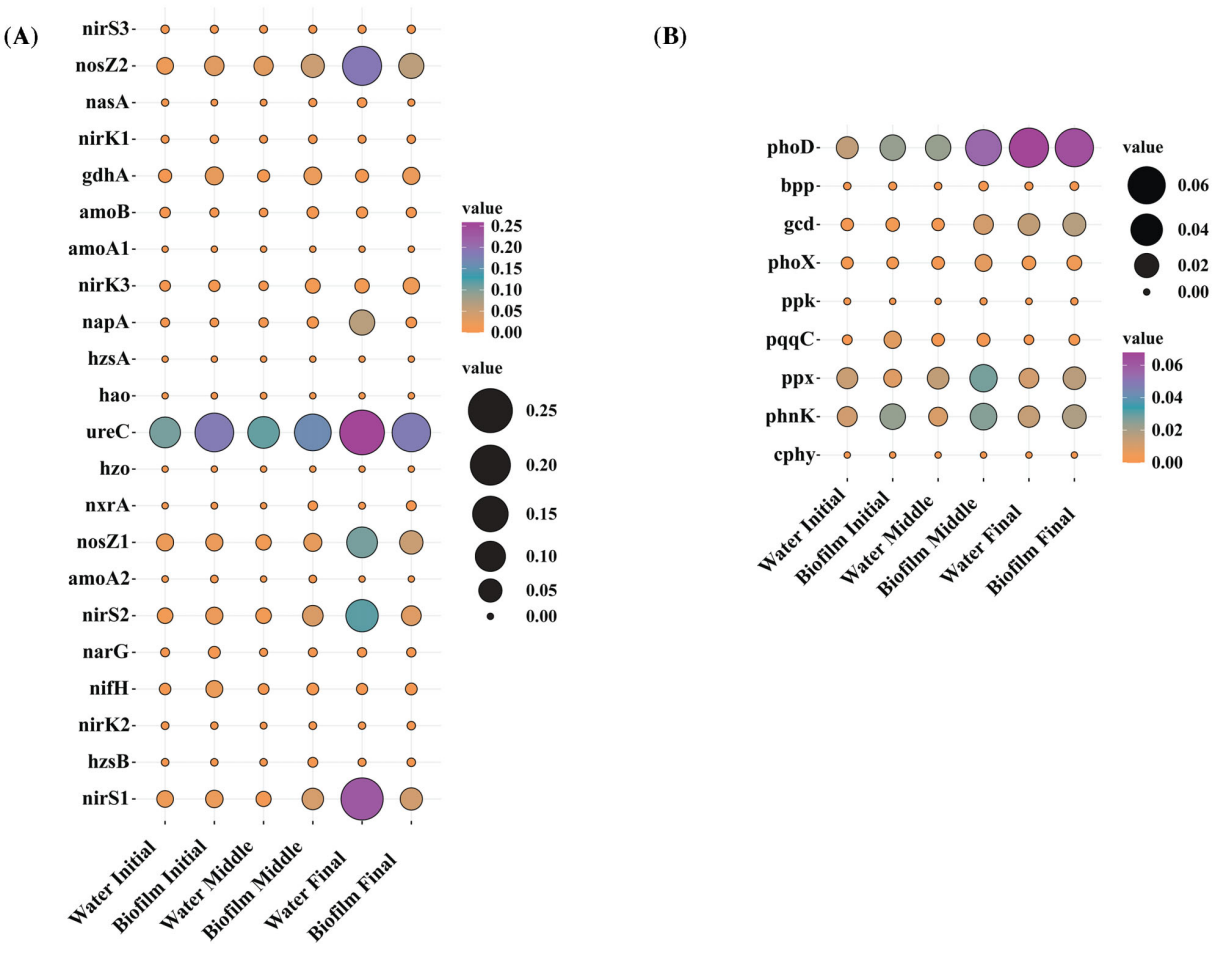
Relative abundance of functional genes involved in nitrogen cycling (A) and phosphorus cycling (B).

Shotgun metagenomic analysis revealed key differences in the relative abundance of nitrogen-cycle genes over three different phases: initial, middle and final (Fig. 3). During the final phase, the relative abundances of *nosZ, napA, nirK/S*, and *nifH* were higher in the water column than in the biofilm. Conversely, the *gdhA* gene showed consistently increase abundance in the biofilm across all three phases, in contrast to the water samples. A marked rise in *ureC* abundance was also observed in the final phase of the water column (Fig. 3A). Regarding phosphorus cycling genes, *ppx* and *phnK* exhibited higher abundances in the biofilm during the middle phase. Additionally, an increase in *phoD* abundance was observed in the final phase of both the water column and biofilm (Fig. 3B).

### Linkages among key genera and functional genes

**Figure 4:**
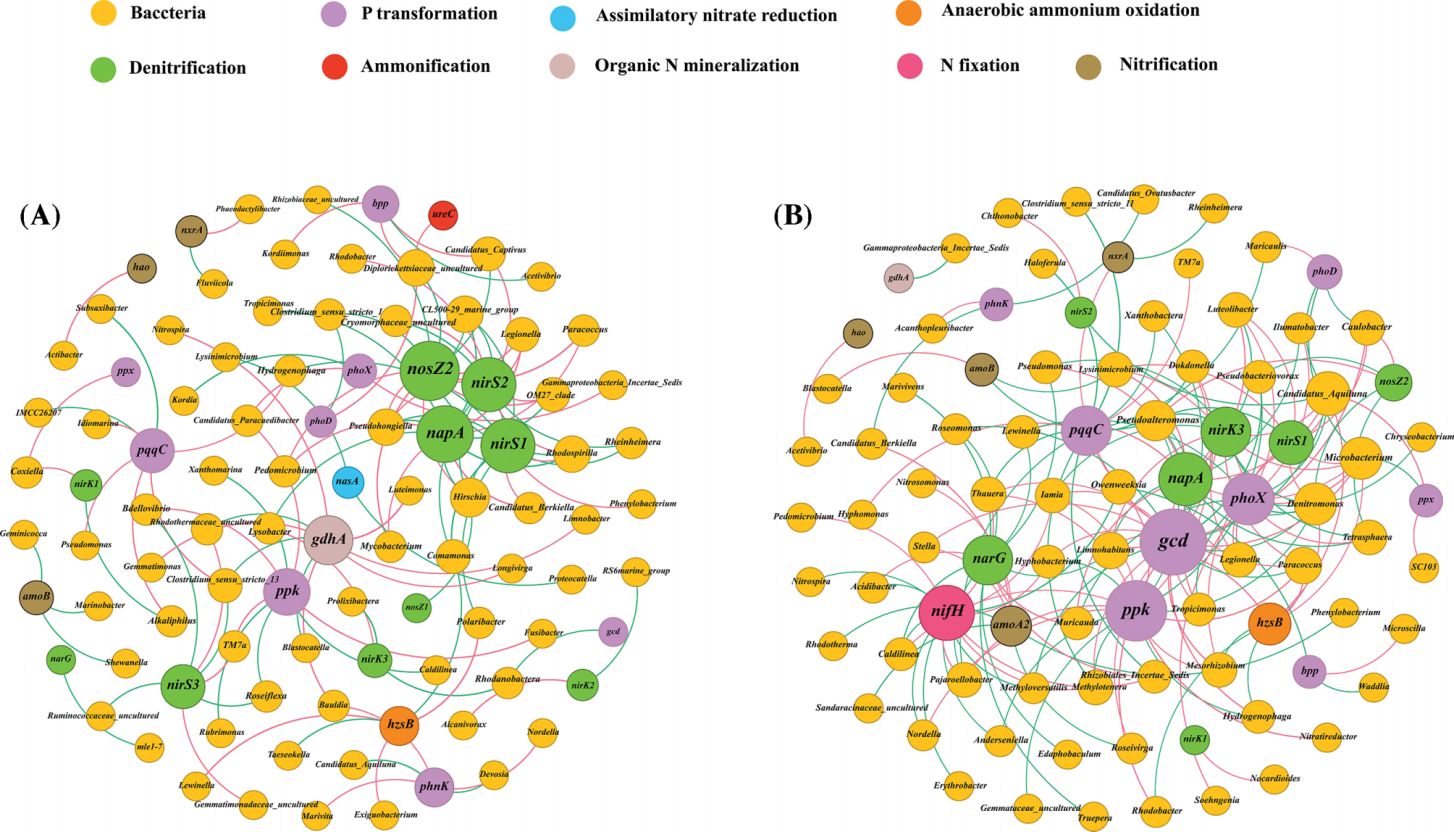
Network analysis depicting correlations between bacterial genera and functional genes in the water column (A) and biofilm (B). Red lines indicate negative correlations, whereas green lines indicate positive correlations.

A network was constructed to analyze the relationship between bacteria and the presence of genes in RAS, revealing strong correlations. The Spearman correlation coefficients for all pairwise interactions were consistently greater than 0.8, with q-values below 0.05, indicating statistically significant associations. The water column network, with a higher number of nodes and edge relationships compared to the biofilm network (Table 1), was notably more complex, highlighting the intricate microbial-genetic interactions within the water column environment. In the water column, the genera *Alkaliphilus, Bdellovibrio, CL500-29, Comamonas, Hirschia, OM27_clade, Pseudohongiella, Rheinheimera*, and *mle1-7* exhibited strong positive correlations with the presence of nitrogen cycling genes *nirKS, nosZ, napA*, and *narG*. Meanwhile, *Candidatus Aquiluna, Rubrimonas, Xanthomarina, Acetivibrio, Kordia*, and *Subsaxibacter* were positively associated with the presence of phosphorus-related genes *phnK, ppk, bpp, phoX*, and *pqqC*. Additionally, *Clostridium sensu stricto 13* showed a positive correlation with the carbon metabolism gene *gdhA*. Both *Comamonas* and *Taeseokella* were positively correlated with the genes *nirS* and *hzsB*, while *Fluviicola* was positively associated with *nxrA*. Finally, *Marinobacter* and *Shewanella* displayed significant positive correlations with the ammonia oxidation gene *amoB* (Fig. 4A). In biofilm, the genera *Denitromonas, Edaphobaculum, Iamia, Limnohabitans, Pseudoalteromonas, Pseudomonas, Marivivens*, and *Tetrasphaera* exhibited positive correlations with the presence of nitrogen cycling genes *nirKS, nosZ, napA*, and *narG*. Additionally, *Candidatus Aquiluna, Denitromonas, Chryseobacterium, Methylotenera, Microbacterium, Pseudobacteriovorax, Clostridium sensu stricto 11, Dokdonella*, and *Haloferula* were positively associated with a suite of genes including *nirS, phoD, gcd, phoX, ppx, ppk*, and *pqqC. Owenweeksia* showed a strong positive correlation with *bpp* and *nifH*, while *Pseudoalteromonas* was positively associated with *gcd, ppk*, and *amoB*. The *Gammaproteobacteria Incertae Sedis* was positively correlated with *gdhA*. Furthermore, *Phenylobacterium, Roseivirga, Lysinimicrobium*, and *Rheinheimera* were positively correlated with *amoAB* and *nxrA*. Finally, *Mesorhizobium* and *Hydrogenophaga* exhibited significant positive correlations with the anammox-related gene *hzsB* (Fig. 4B).

**Table 1:** Key topological parameters statistics of the network.

Groups	Nodes	Edges	Average degree	Modularity	Avg. clustering coefficient	Density	Diameter	Avg. path length
Water	97	117	0.097	0.681	0	0.004	15	5.996
Biofilm	90	169	3.756	0.482	0	0.042	10	3.793

## Discussion

### Dominant bacterial taxa and their contribution to system functionality

RASs have gained increasing attention in shrimp farming due to their ability to conserve water, reduce waste discharge, and maintain stable environmental conditions that support optimal shrimp growth (Dahle et al. 2022). A key feature underpinning the efficiency of RAS is the development of biofilm on system surfaces, which harbors diverse microbial communities responsible for organic matter degradation and nutrient transformation (Chen et al. 2020). In particular, microbial-mediated nitrogen (N) and phosphorus (P) cycling plays a central role in maintaining water quality, with functional genes involved in nitrification and denitrification serving as important indicators of bioremediation potential. In the present study, analysis of bacterial communities across different groups identified Proteobacteria, Actinobacteria, and Bacteroidetes as the predominant phyla in both the water column and biofilm (Emmanuel et al. 2025a), consistent with previous studies reporting similar patterns in which the most prevalent phyla were Proteobacteria and Actinobacteria (Li et al. 2017, 2021, Xie et al. 2020). Notably, Proteobacteria exhibited the highest relative abundance, with a greater enrichment observed in the biofilm compared to the water column. This suggests that biofilm-associated environments provide favorable ecological niches for metabolically versatile taxa. These findings are consistent with previous studies (Lukwambe et al. 2019, Xie et al. 2020), although the relatively higher dominance observed in our system may be attributed to the stable physicochemical conditions maintained in the RAS. The predominance of Proteobacteria in this study, particularly within the biofilm, likely played a significant role in the transformation and removal of nitrogenous compounds. Members of Proteobacteria are key drivers of nitrogen transformation, including nitrification and denitrification, thereby contributing to ammonia removal and overall water quality maintenance (Kleindienst et al. 2014, Raza et al. 2024). Actinobacteria were also consistently detected across all samples, indicating their ecological importance in the system. Their presence may contribute to the decomposition of complex organic compounds and the stabilization of microbial community structure (Li et al. 2015, Zhang et al. 2017, Wang et al. 2018). Meanwhile, Bacteroidetes were more prominently associated with the biofilm, highlighting their role in the degradation of high-molecular-weight organic matter such as proteins and carbohydrates. This functional capacity supports the recycling of nutrients and complements the activity of other microbial groups involved in nitrogen cycling (Gao et al. 2021, Wang et al. 2022). Furthermore, the spatial distribution of microbial taxa between the water column and biofilm underscores the importance of microenvironmental conditions in shaping community structure and function. The biofilm likely provides micro-anaerobic niches that facilitate processes such as denitrification and possibly anammox, as reported in previous studies (Li et al. 2018a, Huang et al. 2020, Wang et al. 2021). In our system, the coexistence of aerobic and anaerobic microenvironments within the biofilm may have enhanced the overall efficiency of nutrient transformation.

In our study, the dominant bacterial classes included Alphaproteobacteria and Gammaproteobacteria (Phylum Proteobacteria), Actinobacteria (Phylum Actinobacteriota), Bacteroidia (Phylum Bacteroidota), and Saccharimonadia (Phylum Patescibacteria) (Fig. 1). Alphaproteobacteria were most abundant in the mid-stage of the water column but showed maximum abundance during the initial phase in the biofilm. Actinobacteria initially increased in both environments but declined over time, whereas Bacteroidia exhibited a marked increase during the final phase in both water and biofilm. Actinobacteria are known to inhibit pathogenic bacteria and aid in nutrient removal (Lukwambe et al. 2015). Both Alphaproteobacteria and Actinobacteria contribute to nitrogen removal, enhance water quality in aquaculture systems, and suppress harmful bacteria (Ghai et al. 2014, Wei et al. 2023). Moreover, ammonia-oxidizing and nitrite-oxidizing bacteria (AOB and NOB), predominantly associated with Alphaproteobacteria, play a critical role in nutrient cycling and are considered key agents of nitrogen removal in wastewater treatment systems (Lukwambe et al. 2015, Zhang et al. 2019, Chang et al. 2022). Collectively, these microbial-mediated processes highlight the central role of community structure in maintaining nutrient balance and supporting stable system functioning in RAS (Emmanuel et al. 2025a). From a production perspective, the observed shrimp growth (0.005–13.89 g over 90 days) appears modest but reflects the controlled operation of RAS, where water quality, stability, and sustainability are prioritized. The results indicate efficient nutrient transformation driven by active microbial processes, supporting system functionality. However, further optimization of feeding, aeration, and stocking strategies is needed to enhance growth performance and improve economic viability (Liang et al. 2025).

### N and P cycle and functional gene distribution

To gain a deeper and more intuitive understanding of nutrient removal efficiency in the systems, this study utilized the GeoChip gene array to measure the absolute quantitative abundance of key genes involved in N and P cycling. The results revealed significant differences between the initial, middle and final groups in terms of gene abundances related to N and P metabolism, highlighting the enhanced nutrient removal performance of the RAS. In our study, the abundance of *ureC*, associated with ammonification, was notably higher in the biofilm compared to water column, particularly in the final phase (Fig. 2). Ammonification is an essential precursor to biological denitrification, as it converts organic nitrogen into ammonium (NH₄⁺), which then undergoes nitrification and subsequent denitrification processes (Wei et al. 2024). In line with this, the nitrification genes *amoA/B*, which encode ammonia monooxygenase, were also enriched in the biofilm compared to water column (Fig. 2), indicating that surface-associated communities play a central role in ammonia oxidation. This is consistent with previous studies showing that nitrification is a highly effective mechanism for removing ammonia and organic pollutants in aquatic systems (Jin et al. 2017). In contrast, genes associated with denitrification exhibited a distinct temporal pattern. The significantly higher abundance of *nosZ* in the final phase of the water column suggests enhanced reduction of nitrous oxide (N₂O) to dinitrogen gas (N₂), indicating active terminal denitrification. This finding is supported by previous studies that emphasize the role of *nosZ* in completing the denitrification process (Lu et al. 2020). Similarly, the increased abundance of *napA* and *nirK/S* further supports the intensification of nitrate and nitrite reduction processes at later stages. The high salinity of seawater, which limits oxygen diffusion, further highlights the importance of biological nitrification and denitrification processes in these systems, particularly under oxygen limiting conditions (Zhang et al. 2017). The nitrogen fixation gene *nifH* showed a contrasting trend, with higher abundance in the biofilm during the initial phase followed by a decline over time. This pattern suggests an early-stage demand for bioavailable nitrogen, which diminishes as internal nitrogen cycling processes become more established and efficient. Such temporal shifts indicate a transition from nitrogen acquisition to nitrogen removal pathways as the system matures. This is in line with previous findings where microbial nitrogen fixation can be outcompeted by other forms of nitrogen processing under varying environmental conditions (Zhao et al. 2019, Tan et al. 2021).

Phosphorus cycling genes also displayed distinct phase and habitat specific patterns. The genes *ppx* and *phnK*, involved in polyphosphate degradation and phosphonate utilization, respectively, exhibited increased absolute abundance in the biofilm during later stages, with peak relative abundance observed in the middle phase (Fig. 3). In bacteria, the *phnK* gene plays a crucial role in encoding the carbon-phosphorus lyase complex, which is essential for converting unactivated phosphonates into phosphate (Yang et al. 2016). The *phnK* also enhances the uptake and utilization of phosphorus-containing compounds via trans membrane transport (Tian et al. 2021). Additionally, the *ppk* and *ppx* genes regulate phosphorus storage and mobilization under aerobic and anaerobic conditions, respectively (Yang et al. 2019). The gene *phoD*, another key component in phosphorus mineralization, shows an increase in both the water column and biofilm during the final phase. The *phoD* gene contributes to the transport and mineralization of organic phosphorus (Willis et al. 2019).

Beyond individual gene patterns, the observed temporal shift from nitrification dominated processes in the early phase to increased denitrification potential in later stages reflects broader system level dynamics inherent to RAS operation (Emmanuel et al. 2025a). The initial enrichment of nitrification genes (*amoA/B*), followed by their relative decline and the concomitant increase in denitrification genes (*nosZ, napA, nirK/S*), is consistent with the progressive accumulation of organic matter associated with increasing biomass and metabolic activity (Yan et al. 2024). Elevated organic carbon availability likely stimulated heterotrophic respiration, leading to localized oxygen depletion and the development of microaerophilic to anoxic conditions that favor denitrification (Zheng et al. 2023). Importantly, as the RAS was operated under relatively stable conditions without major external adjustments, these functional transitions appear to be driven primarily by intrinsic system processes rather than operational interventions. This highlights microbial functional succession as a sensitive indicator of system performance, particularly in relation to organic loading and nutrient transformation efficiency (Emmanuel et al. 2025a). While similar trends have been reported in other aquatic systems (Preheim et al. 2023), the present study provides additional resolution by demonstrating how these dynamics are differentially partitioned between the water column and biofilm, where biofilm communities contribute to early-stage nitrogen retention and transformation, while planktonic communities increasingly facilitate nitrogen removal during later stages. This habitat-specific metabolic partitioning highlights the dynamic interplay between microbial community assembly and environmental conditions in regulating nitrogen cycling efficiency and system stability in RAS.

### Differential abundance of nutrient cycling genes across water and biofilm

The present study revealed clear habitat-specific patterns in the association between bacterial genera and functional genes involved in nitrogen and phosphorus cycling. These patterns not only reflect differences in microbial composition between the water column and biofilm but also highlight distinct ecological strategies governing nutrient transformation processes in each habitat. In the water column, the strongest correlation was observed for *napA* (Fig. 4), which encodes periplasmic nitrate reductase, a key enzyme catalyzing the reduction of nitrate to nitrite in the denitrification pathway (Sparacino-Watkins et al. 2014). Elevated *napA* abundance has been reported in aquatic environments experiencing active nitrate reduction, particularly under microaerophilic or anoxic conditions where periplasmic nitrate reduction confers ecological advantages (Sparacino-Watkins et al. 2014, Zheng et al. 2023). The wide taxonomic range of *napA* associated genera in this study including *Alkaliphilus, Bdellovibrio, Comamonas*, and *Rheinheimera* supports previous findings that denitrification capacity is distributed across phylogenetically diverse lineages rather than restricted to canonical denitrifiers (Yang et al. 2017). Nitrogen cycling in the water column was further supported by strong correlations between denitrification genes *nirK/S, nosZ, narG*, and multiple genera, along with notable associations of *amoB* with *Marinobacter* and *Shewanella*, suggesting active ammonia oxidation potential (Fig. 4) (Zhu et al. 2018, Li et al. 2021). The occurrence of *hzsB* associated taxa *Comamonas* and *Taeseokella* indicates possible contributions from anammox like processes, which have been increasingly recognized outside of Planctomycetes (Trinh et al. 2021). Anammox bacteria utilize ammonia as an electron donor and nitrite as an electron acceptor, producing nitrogen gas and nitrate as end products (Kuenen 2008). In studies utilizing artificial tidal wetland ecosystems for saline water treatment, the *hzsB* gene was found to play a key role in regulating the conversion of ammonium and total nitrogen (Zhang et al. 2023). Collectively, these findings point to a highly interconnected nitrogen cycling network in the water column, characterized by overlapping metabolic capabilities and potential functional redundancy.

Phosphorus cycling in the water column was similarly characterized by diverse gene taxa associations, with genes such as *phnK, ppk, phoX*, and *pqqC* linked to genera including *Candidatus Aquiluna, Rubrimonas*, and *Subsaxibacter*. This distribution reflects the role of Alphaproteobacteria and Bacteroidetes in mediating organic phosphorus turnover, particularly under low to moderate nutrient conditions (Bortolaia et al. 2020). The co-occurrence of multiple phosphorus related genes within the same taxa further suggests coordinated metabolic strategies for nutrient acquisition and recycling in oligotrophic environments. In contrast, biofilm communities exhibited a distinct functional gene profile. The most prominent correlation was observed for *pqqC* (Fig. 4B), encoding pyrroloquinoline quinone synthase, which was involved in redox cofactor biosynthesis and mineral phosphate solubilization. Biofilms are known to enhance extracellular enzyme activity and phosphorus mobilization, with *pqqC* frequently enriched in structured microbial communities where nutrient scavenging is advantageous (Shi et al. 2022). The high association of *pqqC* with multiple genera, including *Candidatus Aquiluna, Methylotenera*, and *Haloferula*, highlights its potential role in sustaining phosphorus turnover within biofilms. Nitrogen cycling in biofilms was supported by strong correlations of *nirK/S, nosZ, napA*, and *narG* with genera such as *Denitromonas, Pseudomonas*, and *Tetrasphaera*, consistent with previous observations that denitrifiers are prevalent and active within biofilm matrices (Qu et al. 2021). Additionally, the associations of *nifH* with *Owenweeksia* suggests possible nitrogen fixation activity, while *hzsB* correlation with *Mesorhizobium* and *Hydrogenophaga* indicate potential anammox-related processes. The detection of *amoA/B* and *nxrA* correlations points to complete nitrification potential within biofilms, possibly driven by *Nitrospira* like comammox organisms and ammonia-oxidizing Proteobacteria (Vijayan et al. 2021). These findings imply that biofilms provide spatially heterogeneous niches that support the coupling of aerobic and anaerobic processes, thereby enhancing overall nutrient transformation efficiency. Importantly, network analysis revealed marked differences in complexity between the two habitats. The water column network exhibited higher connectivity, with a greater number of gene taxa interactions and more diffuse associations across multiple functional groups. This elevated complexity suggests increased functional redundancy and metabolic flexibility, which may confer resilience to environmental fluctuations and support dynamic nutrient processing (González-Motos et al. 2025). In contrast, the biofilm network displayed a more modular and tightly organized structure, reflecting niche partitioning and specialization driven by surface-associated growth and microscale gradients in oxygen and nutrient availability. Such organization likely promotes efficient resource utilization and stable system performance over time (Schoina et al. 2022). Taken together, these findings demonstrate that network complexity and functional gene distribution are strongly shaped by habitat-specific conditions in RAS. The highly interconnected and flexible microbial networks in the water column complement the structured and efficient biofilm communities, collectively contributing to robust and sustained nutrient cycling (Schoina et al. 2022). This integrative perspective advances our understanding of how microbial interactions govern ecosystem functioning and provides a mechanistic basis for optimizing microbial management strategies in engineered aquaculture systems (Emmanuel et al. 2025a).

## Conclusion

This study demonstrates that bacterial community succession in RAS is both phase dependent and habitat specific, with distinct yet complementary roles for water column and biofilm communities in nutrient cycling. Alphaproteobacteria, Actinobacteria, Bacteroidia, and Gammaproteobacteria dominated across phases, but their abundance patterns reflected differing ecological strategies between water column and biofilm. Functional gene analysis further revealed that denitrification genes were enriched in the water column during the final phase, whereas nitrogen fixation and organic nitrogen mineralization were more prominent in early-stage biofilms, with phosphorus metabolism genes increasing in later stages across both habitats. Importantly, these findings advance current understanding of microbial community dynamics in RAS by linking taxonomic composition with functional potential across spatial and temporal gradients. The observed habitat-specific partitioning of microbial processes provides new insight into how microbial interactions drive nutrient transformation and system stability. Additionally, network analysis indicating more complex microbial gene associations in the water column suggests a higher degree of functional redundancy and resilience in planktonic communities. From an applied perspective, this study offers valuable guidance for optimizing RAS performance through targeted management of microbial communities. By understanding the functional roles of key taxa and genes, strategies can be developed to enhance nitrogen and phosphorus removal, improve water quality, and promote sustainable aquaculture practices. Collectively, this work contributes to the broader field by providing a mechanistic framework for integrating microbial ecology into the design and management of efficient, environmentally sustainable aquaculture systems.

## Supplementary Material

fiag081_Supplemental_Files

## References

[bib1] Ahmed N, Turchini GM. Recirculating aquaculture systems (RAS): environmental solution and climate change adaptation. J Cleaner Prod. 2021;297:126604. 10.1016/j.jclepro.2021.126604

[bib2] Bartelme RP, McLellan SL, Newton RJ. Freshwater recirculating aquaculture system operations drive biofilter bacterial community shifts around a stable nitrifying consortium of ammonia-oxidizing archaea and comammox Nitrospira. Front Microbiol. 2017;8:101. 10.3389/fmicb.2017.00101 28194147 PMC5276851

[bib3] Bortolaia V, Kaas RS, Ruppe E et al. ResFinder 4.0 for predictions of phenotypes from genotypes. J Antimicrob Chemother. 2020;75:3491–500. 10.1093/jac/dkaa345 32780112 PMC7662176

[bib4] Camargo JA, Alonso Á. Ecological and toxicological effects of inorganic nitrogen pollution in aquatic ecosystems: a global assessment. Environ Int. 2006;32:831–49. 10.1016/j.envint.2006.05.002 16781774

[bib5] Chang F, He S, Dang C. Assisted selection of biomarkers by linear discriminant analysis effect size (LEfSe) in microbiome data. J Vis Exp. 2022;183:e61715. 10.3791/61715 35635468

[bib6] Chen Z, Chang Z, Zhang L et al. Effects of carbon source addition on microbial community and water quality in recirculating aquaculture systems for Litopenaeus vannamei. Fish Sci. 2020;86:507–17. 10.1007/s12562-020-01423-3

[bib7] Dahle SW, Attramadal KJK, Vadstein O et al. Microbial community dynamics in a commercial RAS for production of Atlantic salmon fry (Salmo salar). Aquaculture. 2022;546:737382. 10.1016/j.aquaculture.2021.737382

[bib8] Daims H, Lebedeva EV, Pjevac P et al. Complete nitrification by Nitrospira bacteria. Nature. 2015;528:504–9. 10.1038/nature16461 26610024 PMC5152751

[bib9] Daims H, Lücker S, Wagner M. A new perspective on microbes formerly known as nitrite-oxidizing bacteria. Trends Microbiol. 2016;24:699–712. 10.1016/j.tim.2016.05.004 27283264 PMC6884419

[bib10] Deng Y, He Z, Xiong J et al. Elevated carbon dioxide accelerates the spatial turnover of soil microbial communities. Global Change Biol. 2016;22:957–64. 10.1111/gcb.13098 26414247

[bib11] Emmanuel A, Raza B, Ramzan MN et al. Bacterial community of the shrimp (Litopenaeus vannamei) gut and its relationship with water quality in a recirculating aquaculture system (RAS). Aquacult Int. 2025b;33:640. 10.1007/s10499-025-02349-2

[bib12] Emmanuel A, Wei Y, Ramzan MN et al. Dynamics of bacterial communities and their relationship with nutrients in a full-scale shrimp recirculating aquaculture system in brackish water. Animals. 2025a;15:1400. 10.3390/ani15101400 40427277 PMC12108446

[bib13] Feng W, Liang J, Hale LE et al. Enhanced decomposition of stable soil organic carbon and microbial catabolic potentials by long-term field warming. Global Change Biol. 2017;23:4765–76. 10.1111/gcb.13755 28597589

[bib14] Gao T, Li H, He Y et al. The variations of bacterial community structures in tailing soils suffering from heavy metal contaminations. Water Air Soil Pollut. 2021;232:1–17. 10.1007/s11270-021-05338-2

[bib15] Ghai R, Mizuno CM, Picazo A et al. Key roles for freshwater Actinobacteria revealed by deep metagenomic sequencing. Mol Ecol. 2014;23:6073–90. 10.1111/mec.12985 25355242

[bib16] González-Motos S, Montiel L, Balagué V et al. Functional redundancy enables emergent metabolic dynamics in marine microbiomes. Biorxiv. 2025;8. 10.1101/2025.08.12.669827

[bib17] Graham EB, Knelman JE, Schindlbacher A et al. Microbes as engines of ecosystem function: when does community structure enhance predictions of ecosystem processes?. Front Microbiol. 2016;7:214. 10.3389/fmicb.2016.00214 26941732 PMC4764795

[bib18] Hazen TC, Dubinsky EA, DeSantis TZ et al. Deep-sea oil plume enriches indigenous oil-degrading bacteria. Science. 2010;330:204–8. 10.1126/science.1195979 20736401

[bib19] He Z, Deng Y, Van Nostrand JD et al. GeoChip 3.0 as a high throughput tool for analyzing microbial community composition, structure and functional activity. ISME J. 2010;4:1167–79. 10.1038/ismej.2010.46 20428223

[bib20] Huang F, Pan L, He Z et al. Identification, interactions, nitrogen removal pathways and performances of culturable heterotrophic nitrification–aerobic denitrification bacteria from mariculture water by using cell culture and metagenomics. Sci Total Environ. 2020;732:139268. 10.1016/j.scitotenv.2020.139268 32402929

[bib21] Huang Z, Jiang Y, Song X et al. Ammonia-oxidizing bacteria and archaea within biofilters of a commercial recirculating marine aquaculture system. AMB Expr. 2018;8:17. 10.1186/s13568-018-0551-1 PMC5810308 29429071

[bib22] Hüpeden J, Wegen S, Off S et al. Relative abundance of Nitrotoga spp. in a biofilter of a cold-freshwater aquaculture plant appears to be stimulated by slightly acidic pH. Appl Environ Microb. 2016;82:1838–45. 10.1128/AEM.03163-15 PMC4784051 26746710

[bib23] Jin Q, Lu J, Wu J et al. Simultaneous removal of organic carbon and nitrogen pollutants in the Yangtze estuarine sediment: the role of heterotrophic nitrifiers. Estuarine Coastal Shelf Sci. 2017;191:150–6. 10.1016/j.ecss.2017.04.019

[bib24] Keuter S, Beth S, Quantz G et al. Long-term monitoring of nitrification and nitrifying communities during biofilter activation of two marine recirculation aquaculture systems (RAS). Int J Aquac Fish Sci. 2017;3:051–61. 10.17352/2455-8400.000029

[bib25] Kim K, Hur JW, Kim S et al. Biological wastewater treatment: comparison of heterotrophs (BFT) with autotrophs (ABFT) in aquaculture systems. Bioresour Technol. 2020;296:122293. 10.1016/j.biortech.2019.122293 31677407

[bib26] Kleindienst S, Herbst FA, Stagars M et al. Diverse sulfate-reducing bacteria of the Desulfosarcina/Desulfococcus clade are the key alkane degraders at marine seeps. ISME J. 2014;8:2029–44. 10.1038/ismej.2014.51 24722631 PMC4184016

[bib27] Kuenen JG . Anammox bacteria: from discovery to application. Nat Rev Micro. 2008;6:320–6. 10.1038/nrmicro1857 18340342

[bib28] Li D, Liu CM, Luo R et al. MEGAHIT: an ultra-fast single-node solution for large and complex metagenomics assembly via succinct de Bruijn graph. Bioinformatics. 2015;31:1674–6. 10.1093/bioinformatics/btv033 25609793

[bib29] Li H, Cui Z, Cui H et al. Hazardous substances and their removal in recirculating aquaculture systems: a review. Aquaculture. 2023;569:739399. 10.1016/j.aquaculture.2023.739399

[bib30] Li K, Hu J, Li T et al. Microbial abundance and diversity investigations along rivers: current knowledge and future directions. WIREs Water. 2021;8:e1547. 10.1002/wat2.1547

[bib31] Li M, Liang Z, Callier MD et al. Nitrogen and organic matter removal and enzyme activities in constructed wetlands operated under different hydraulic operating regimes. Aquaculture. 2018a;496:247–54. 10.1016/j.aquaculture.2018.06.016

[bib32] Li X, Meng D, Li J et al. Response of soil microbial communities and microbial interactions to long-term heavy metal contamination. Environ Pollut. 2017;231:908–17. 10.1016/j.envpol.2017.08.057 28886536

[bib33] Liang Q, Liu G, Luan Y et al. Impact of feeding frequency on growth performance and antioxidant capacity of Litopenaeus vannamei in recirculating aquaculture systems. Animals. 2025;15:192. 10.3390/ani15020192 39858192 PMC11758656

[bib34] Lu J, Zhang Y, Wu J et al. Nitrogen removal in recirculating aquaculture water with high dissolved oxygen conditions using the simultaneous partial nitrification, anammox and denitrification system. Bioresour Technol. 2020;305:123037. 10.1016/j.biortech.2020.123037 32105846

[bib35] Lukwambe B, Qiuqian L, Wu J et al. The effects of commercial microbial agents (probiotics) on phytoplankton community structure in intensive white shrimp (Litopenaeus vannamei) aquaculture ponds. Aquacult Int. 2015;23:1443–55. 10.1007/s10499-015-9895-6

[bib36] Lukwambe B, Zhao L, Nicholaus R et al. Bacterioplankton community in response to biological filters (clam, biofilm, and macrophytes) in an integrated aquaculture wastewater bioremediation system. Environ Pollut. 2019;254:113035. 10.1016/j.envpol.2019.113035 31421576

[bib37] Mardis E, McCombie WR. Library quantification using SYBR green-quantitative polymerase chain reaction (qPCR). Cold Spring Harb Protoc. 2017; 2017:pdb.prot094714. 10.1101/pdb.prot094714 27803268

[bib38] Ngugi DK, Blom J, Stepanauskas R et al. Diversification and niche adaptations of Nitrospina-like bacteria in the polyextreme interfaces of Red Sea brines. ISME J. 2016;10:1383–99. 10.1038/ismej.2015.214 26657763 PMC5029188

[bib39] Nolan T, Hands RE, Bustin SA. Quantification of mRNA using real-time RT PCR. Nat Protoc. 2006;1:1559–82. 10.1038/nprot.2006.236 17406449

[bib40] Penuelas J, Poulter B, Sardans J et al. Human-induced nitrogen-phosphorus imbalances alter natural and managed ecosystems across the globe. Nat Commun. 2013;4:2934. 10.1038/ncomms3934 24343268

[bib41] Petersen DG, Blazewicz SJ, Firestone M et al. Abundance of microbial genes associated with nitrogen cycling as indices of biogeochemical process rates across a vegetation gradient in Alaska. Environ Microbiol. 2012;14:993–1008. 10.1111/j.1462-2920.2011.02679.x 22225623

[bib42] Preheim S, Morris S, Zhang Y et al. Major trends and environmental correlates of spatiotemporal shifts in the distribution of genes compared to a biogeochemical model simulation in the Chesapeake Bay. Biorxiv. 2023:2023–01.

[bib43] Qu J, Yang H, Liu Y et al. The study of natural biofilm formation and microbial community structure for recirculating aquaculture system. IOP Conf Ser: Earth Environ Sci. 2021;742:012018. 10.1088/1755-1315/742/1/012018

[bib44] Ramzan MN, Shen D, Wei Y et al. Performance and microbial community analysis of integrated bioremediation systems with photosynthetic bacteria in treating mariculture tailwater. J Ocean Univ China. 2025a;24:515–24. 10.1007/s11802-025-5889-9

[bib45] Ramzan MN, Shen D, Wei Y et al. Nitrogen- and phosphorus-related functional genes enhance nutrient removal in the integrated aquaculture wastewater bioremediation system in the presence of photosynthetic bacteria. Aquacult Int. 2025b;33:131. 10.1007/s10499-024-01809-5

[bib46] Raza B, Zheng Z, Zhu J et al. A review: microbes and their effect on growth performance of Litopenaeus vannamei (white leg shrimp) during culture in biofloc technology system. Microorganisms. 2024;12:1013. 10.3390/microorganisms12051013 38792842 PMC11123971

[bib47] Ruiz P, Vidal JM, Sepúlveda D et al. Overview and future perspectives of nitrifying bacteria on biofilters for recirculating aquaculture systems. Rev Aquac. 2019;12:1478–94. 10.1111/raq.12392

[bib48] Schoina E, Doulgeraki AI, Miliou H et al. Dynamics of Water and Biofilm Bacterial Community Composition in a Mediterranean Recirculation Aquaculture System. Aquaculture J. 2022;2:164–79. https://www.mdpi.com/2673-9496/2/2/8

[bib49] Shao S, Hu Y, Cheng J et al. Action of oxytetracycline (OTC) degrading bacterium and its application in moving bed biofilm reactor (MBBR) for aquaculture wastewater pre-treatment. Ecotoxicol Environ Saf. 2019;171:833–42. 10.1016/j.ecoenv.2019.01.040 30660977

[bib50] Shi W, Xing Y, Zhu Y et al. Diverse responses of pqqC- and phoD-harbouring bacterial communities to variation in soil properties of Moso bamboo forests. Microb Biotechnol. 2022;15:2097–111. 10.1111/1751-7915.14029 35298867 PMC9249317

[bib51] Sparacino-Watkins C, Stolz JF, Basu P. Nitrate and periplasmic nitrate reductases. Chem Soc Rev. 2014;43:676–706. 10.1039/C3CS60249D 24141308 PMC4080430

[bib52] Tan X, Yang YL, Liu YW et al. The synergy of porous substrates and functional genera for efficient nutrients removal at low temperature in a pilot-scale two-stage tidal flow constructed wetland. Bioresour Technol. 2021;319. 10.1016/j.biortech.2020.124135 32979599

[bib53] Tian J, Kuang X, Tang M et al. Biochar application under low phosphorus input promotes soil organic phosphorus mineralization by shifting bacterial phoD gene community composition. Sci Total Environ. 2021;779:146556. 10.1016/j.scitotenv.2021.146556 34030240

[bib54] Trinh HP, Lee SH, Jeong G et al. Recent developments of the mainstream anammox processes: challenges and opportunities. J Environ Chem Eng. 2021;9:105583. 10.1016/j.jece.2021.105583

[bib55] Tu Q, Yu H, He Z et al. GeoChip 4: a functional gene-array-based high-throughput environmental technology for microbial community analysis. Mol Ecol Resour. 2014;14:914–28. 10.1111/1755-0998.12239 24520909

[bib56] van der Heijden MGA, Bardgett RD, van Straalen NM. The unseen majority: soil microbes as drivers of plant diversity and productivity in terrestrial ecosystems. Ecol Lett. 2008;11:296–310. 10.1111/j.1461-0248.2007.01139.x 18047587

[bib57] van Kessel MAHJ, Harhangi HR, van de Pas-Schoonen K et al. Biodiversity of N-cycle bacteria in nitrogen removing moving bed biofilters for freshwater recirculating aquaculture systems. Aquaculture. 2010;306:177–84. 10.1016/j.aquaculture.2010.05.019

[bib58] van Kessel MAHJ, Speth DR, Albertsen M et al. Complete nitrification by a single microorganism. Nature. 2015;528:555–9. 10.1038/nature16459 26610025 PMC4878690

[bib59] Vanwonterghem I, Jensen PD, Ho DP et al. Linking microbial community structure, interactions and function in anaerobic digesters using new molecular techniques. Curr Opin Biotechnol. 2014;27:55–64. 10.1016/j.copbio.2013.11.004 24863897

[bib60] Vijayan A, Vattiringal Jayadradhan RK, Pillai D et al. Nitrospira as versatile nitrifiers: taxonomy, ecophysiology, genome characteristics, growth, and metabolic diversity. J Basic Microbiol. 2021;61:88–109. 10.1002/jobm.202000485 33448079

[bib61] Wang J, Lu J, Zhang Y et al. Metagenomic analysis of antibiotic resistance in coastal industrial mariculture systems. Bioresour Technol. 2018b;253:235–43. 10.1016/j.biortech.2018.01.035 29353751

[bib62] Wang L, Sun J, Zheng W et al. Effects of a combined biological restoration technology on nitrogen and phosphorus removal from eutrophic water. Pol J Environ Stud. 2018;27:2293–301. 10.15244/pjoes/77609

[bib63] Wang Q, Fu W, Lu R et al. Characterization of Bacillus subtilis Ab03 for efficient ammonia nitrogen removal. Syst Microbiol and Biomanuf. 2022;2:580–8. 10.1007/s43393-022-00088-6

[bib64] Wang Z, Gao J, Dai H et al. Microplastics affect the ammonia oxidation performance of aerobic granular sludge and enrich the intracellular and extracellular antibiotic resistance genes. J Hazard Mater. 2021;409:124981. 10.1016/j.jhazmat.2020.124981 33387747

[bib65] Wei J, Huang X, Wang H et al. Insight into biofilm formation of wastewater treatment processes: nitrogen removal performance and biological mechanisms. Sci Total Environ. 2023;903:166550. 10.1016/j.scitotenv.2023.166550 37633400

[bib66] Wei Y, Shen D, Nicholaus R et al. Exogenous compound bacteria enhance the nutrient removal efficiency of integrated bioremediation systems: functional genes and microorganisms play key roles. Environ Res. 2024;252:118864. 10.1016/j.envres.2024.118864 38574987

[bib67] Weinstock GM . Genomic approaches to studying the human microbiota. Nature. 2012;489:250–6. 10.1038/nature11553 22972298 PMC3665339

[bib68] Willis A, Chuang AW, Dyhrman S et al. Differential expression of phosphorus acquisition genes in response to phosphorus stress in two Raphidiopsis raciborskii strains. Harmful Algae. 2019;82:19–25. 10.1016/j.hal.2018.12.003 30928007

[bib69] Wuchter C, Abbas B, Coolen MJL et al. Archaeal nitrification in the ocean. Proc Natl Acad Sci USA. 2006;103:12317–22. 10.1073/pnas.0600756103 16894176 PMC1533803

[bib70] Xie B, Tang X, Ng HY et al. Biological sulfamethoxazole degradation along with anaerobically digested centrate treatment by immobilized microalgal bacterial consortium: performance, mechanism and shifts in bacterial and microalgal communities. Chem Eng J. 2020;388:124217. 10.1016/j.cej.2020.124217

[bib71] Yan Y, Zhou J, Du C et al. Relationship between nitrogen dynamics and key microbial nitrogen-cycling genes in an intensive freshwater aquaculture pond. Microorganisms. 2024;12:266. 10.3390/microorganisms12020266 38399670 PMC10892730

[bib72] Yang K, Ren Z, Raushel FM et al. Structures of the carbon-phosphorus lyase complex reveal the binding mode of the NBD-like PhnK. Structure. 2016;24:37–42. 10.1016/j.str.2015.11.009 26724995 PMC4706772

[bib73] Yang L, Wang XH, Cui S et al. Simultaneous removal of nitrogen and phosphorous by heterotrophic nitrification–aerobic denitrification of a metal resistant bacterium Pseudomonas putida strain NP5. Bioresour Technol. 2019;285:121360. 10.1016/j.biortech.2019.121360 31015182

[bib74] Yang Y, Xie L, Tao X et al. Municipal wastewater treatment by the bioaugmentation of Bacillus sp. K5 within a sequencing batch reactor. PLoS One. 2017;12:e0178837. 10.1371/journal.pone.0178837 28594856 PMC5464570

[bib75] Zarraonaindia I, Smith DP, Gilbert JA. Beyond the genome: community-level analysis of the microbial world. Biol Philos. 2013;28:261–82. 10.1007/s10539-012-9357-8 23482824 PMC3585761

[bib76] Zhang H, He H, Chen S et al. Abundance of antibiotic resistance genes and their association with bacterial communities in activated sludge of wastewater treatment plants: geographical distribution and network analysis. J Environ Sci. 2019;82:24–38. 10.1016/j.jes.2019.02.023 31133267

[bib77] Zhang M, Sun S, Gu X et al. Efficient nitrogen removal pathways and corresponding microbial evidence in tidal flow constructed wetlands for saline water treatment. Environ Res. 2023;234:116548. 10.1016/j.envres.2023.116548 37414392

[bib78] Zhang Y, Jiang WL, Xu RX et al. Effect of short-term salinity shock on unacclimated activated sludge with pressurized aeration in a sequencing batch reactor. Sep Purif Technol. 2017;178:200–6. 10.1016/j.seppur.2017.01.048

[bib79] Zhao Y, Liu D, Huang W et al. Insights into biofilm carriers for biological wastewater treatment processes: current state-of-the-art, challenges, and opportunities. Bioresour Technol. 2019;288:121619. 10.1016/j.biortech.2019.121619 31202712

[bib80] Zheng X, Yan Z, Zhao C et al. Homogeneous environmental selection mainly determines the denitrifying bacterial community in intensive aquaculture water. Front Microbiol. 2023;14:1280450. 10.3389/fmicb.2023.1280450 38029183 PMC10653326

[bib81] Zhou J, He Z, Yang Y et al. Alvarez-Cohen L. High-throughput metagenomic technologies for complex microbial community analysis: open and closed formats. mBio. 2015;6:e03388–14. 10.1128/mBio.02288-14 PMC4324309 25626903

[bib82] Zhu W, Wang C, Hill J et al. A missing link in the estuarine nitrogen cycle? Coupled nitrification–denitrification mediated by suspended particulate matter. Sci Rep. 2018;8:2282. 10.1038/s41598-018-20688-4 29396528 PMC5797115

