# Supplementary material for: Bacterial community succession and functional gene dynamics of nitrogen and phosphorus cycling in recirculating aquaculture systems of shrimp
Source: FEMS Microbiol Ecol. 2026 Jul 25;102(8):fiag081. doi: 10.1093/femsec/fiag081 (PMC13421783; doi:10.1093/femsec/fiag081)
Supplement: fiag081_Supplemental_Files [file fiag081_supplemental_files.zip › Supplementary files.docx]

**Appendix A**

**Table A.1 Growth performance of shrimp in RAS**

| Initial  ________________________________­­­­­­­­­­­­­ | | | | | Final  ________________________________________ | | | |
| --- | --- | --- | --- | --- | --- | --- | --- | --- |
|  | Stocking density (ind m^−3^) | Weight (g) | Number of shrimps per tank | Total weight (kg) per tank | Stocking density (kg m^−3^) | Weight (g) | Number of shrimps per tank | Total weight (kg) per tank |
| RAS | 658 ± 0.0032 | 0.02 ± 0.005 | 25000 | 0.5 ± 0.125 | 9.14 ± 1.53 | 13.89 ± 2.71 | 18,053 | 250.7 ± 48.92 |

**Table A.2 The percentages of shrimp feed ingredients over cultivation.**

| Ingredients | Postlarvae (length <3cm) | Juveniles  (length 3~6cm) | Adults  (length>6cm) |
| --- | --- | --- | --- |
| Crude protein | 48.0 | 43.0 | 43.0 |
| Crude fat | 6.0 | 6.0 | 6.0 |
| Crude fibre | 4.0 | 6.0 | 6.0 |
| Crude ash | 17.0 | 18.0 | 18.0 |
| Total phosphate | 1.4 | 1.0 | 1.0 |
| Lysine | 3.2 | 2.3 | 2.4 |
| water | 10.0 | 12.0 | 12.0 |

**Table A.3 Shrimp growth performance indicators**

| Parameters | Value | Remarks |
| --- | --- | --- |
| Daily Growth Rate | 0.154 g | Per day |
| Survival Rate | 72.21 % | At harvesting |
| Specific Growth Rate (SGR) | 7.27 % | Per day based on log weight change |
| Initial Biomass | 0.5 kg | Per tank |
| Final Biomass | 250.7 kg | Per tank |
| Weight Gain (Biomass) | 250.2 kg | Per tank |
| Total Feed Given | 452.16 kg | Estimated over 90 days |
| Feed Conversion Ratio (FCR) | 1.81 | Feed used per kg weight gain |
| Protein in Feed | 48 % | Commercial pellet composition |
| Protein Intake | 216.93 kg | 48% of total feed |
| Protein Efficiency Ratio (PER) | 1.15 | Weight gain per kg protein intake |

**Table A.4 Quantitative effects of sampling time and environmental factors on variation in bacterial community structure using one-way ANOVA (**p* < 0.05, ** *p* < 0.01, ****p* < 0.001).**

|  | Days  ___________ | | Factors  ___________ | | Days*Factors  ___________ | |
| --- | --- | --- | --- | --- | --- | --- |
|  | F | P | F | P | F | P |
| Community composition | 109.4 | 0.000 | 180.2 | 0.000 | 18.6 | 0.000 |

**Appendix B**

**
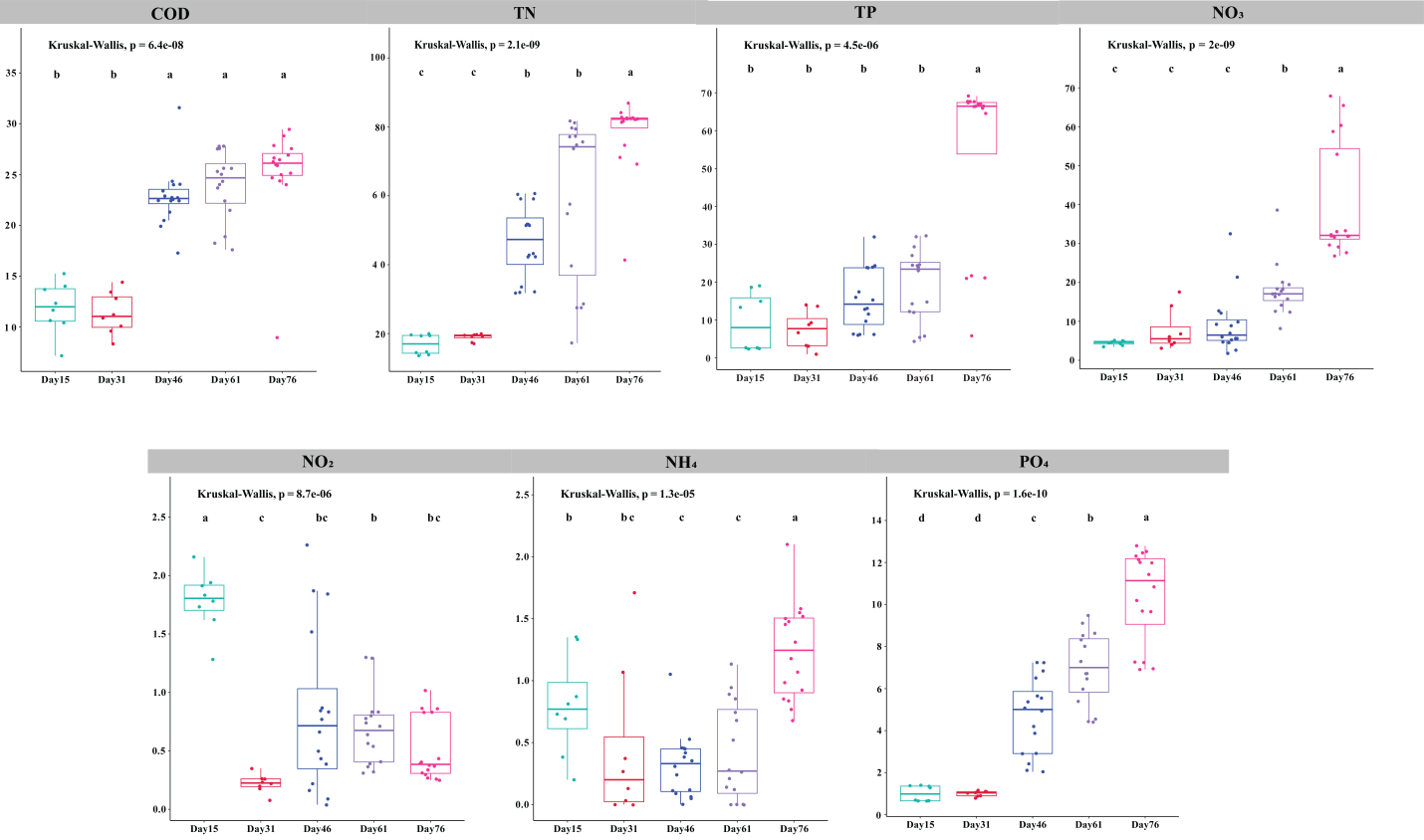
**

Figure A1: Nutrient content of water at different time. Total nitrogen (TN), Total phosphorus (TP), Chemical oxygen demand (COD_Mn_), Ammonia nitrogen (NH_4_^+^ -N), Nitrate (NO_3_^-^ -N), Nitrite (NO_2_^-^ -N), and Orthophosphate (PO_4_^3-^ -P). Different letters specify the significant difference between groups (P < 0.05); same letters represent no significant difference.
